# Common and Uncommon Errors in Emergency Ultrasound

**DOI:** 10.3390/diagnostics12030631

**Published:** 2022-03-04

**Authors:** Marco Di Serafino, Francesca Iacobellis, Maria Laura Schillirò, Divina D’auria, Francesco Verde, Dario Grimaldi, Giuseppina Dell’Aversano Orabona, Martina Caruso, Vittorio Sabatino, Chiara Rinaldo, Pasquale Guerriero, Vito Cantisani, Gianfranco Vallone, Luigia Romano

**Affiliations:** 1 Department of General and Emergency Radiology, “Antonio Cardarelli” Hospital, 80131 Naples, Italy; iacobellisf@gmail.com (F.I.); marialaura.schilliro@gmail.com (M.L.S.); francescoverde87@gmail.com (F.V.); dariogrimaldi@me.com (D.G.); giuseppinadellaversanoorabona@gmail.com (G.D.O.); caruso.martina90@gmail.com (M.C.); vittorio.sabatino@gmail.com (V.S.); chiara_rinaldo@libero.it (C.R.); luigia.romano1@gmail.com (L.R.); 2 Department of Advanced Biomedical Sciences, University of Naples “Federico II”, 80131 Naples, Italy; divinadauria@gmail.com; 3 Department of Life and Health, University of Molise “V. Tiberio”, 86100 Campobasso, Italy; pasquale.guerriero@unimol.it (P.G.); gianfranco.vallone1@gmail.com (G.V.); 4 Department of Radiology, Sapienza Rome University, Policlinico Umberto I, 00185 Rome, Italy; vito.cantisani@uniroma1.it

**Keywords:** ultrasonography, B-mode, artifacts, diagnostic mistakes, diagnostic pitfalls, abdominal trauma, emergency

## Abstract

Errors in emergency ultrasound (US) have been representing an increasing problem in recent years thanks to several unique features related to both the inherent characteristics of the discipline and to the latest developments, which every medical operator should be aware of. Because of the subjective nature of the interpretation of emergency US findings, it is more prone to errors than other diagnostic imaging modalities. The misinterpretation of US images should therefore be considered as a serious risk in diagnosis. The etiology of error is multi-factorial: it depends on environmental factors, patients and the technical skills of the operator; it is influenced by intrinsic US artifacts, poor clinical correlation, US-setting errors and anatomical variants; and it is conditioned by the lack of a methodologically correct clinical approach and excessive diagnostic confidence too. In this review, we evaluate the common and uncommon sources of diagnostic errors in emergency US during clinical practice, showing how to recognize and avoid them.

## 1. Introduction

Ultrasound (US) is a routine imaging procedure that is frequently the initial course of action for emergency care and plays a critical role in the diagnosis of patients in the emergency room. Because of the subjective nature of the interpretation of emergency US findings, it is more prone to errors than other diagnostic imaging modalities. The misinterpretation of US images should therefore be considered as a serious risk in diagnosis [1]. Furthermore, the emergency room setting is an environment with a particular risk for malpractice claims [1,2].

## 2. Findings

US diagnostic errors in the emergency setting can be classified into three groups as follows:

Errors dependent on:

1. Environmental factors;
2. Patients;
3. Technical skills of the operator.

### 2.1. Environmental Factors

A common malpractice claim scenario takes place in an emergency room. Overcrowding in emergency rooms, a large number of investigations of differing appropriateness, and quick diagnosis and management all contribute to a high-risk setting. These variables can affect the quality of US examinations, by putting the medical operator under stress [1,2] when performing US and interpreting results.

### 2.2. Patients

Patients in the emergency room are unlikely to be adequately prepared for unanticipated US examinations, such as fasting status and bladder distention. Some patients may be unwilling to cooperate, suffer extreme pain from the probe’s pressure, or be under the influence of alcohol or drugs. These factors can complicate the performance of the US examination, especially by providing a poor panoramic view [1,2].

### 2.3. The Technical Skills of the Operator

The accuracy of the US examination is directly related to the operator’s skill, training, and experience. The medical operator’s responsibilities include fully exploiting the diagnostic capability of US, knowing what to look for, and having the competence to interpret the findings based on understanding the physiology and pathological changes of the examined organs [1,2]. Furthermore, the interpretation of US examinations requires a subjective judgement that is heavily influenced by the operator and cannot be achieved only on the basis of the images. Unlike other imaging procedures, US examination is a real-time test that cannot be delegated, although in many countries across Europe and North America it is usually performed by a specialized non-medical technical operator such as a sonographer, followed by a delayed report by a physician; in this way it loses its special value—facilitating real-time imaging. The increased number of devices and operators devoted to US influence a large number of examinations; interpretational doubts create the need for further diagnostic imaging confirmations and a consequent increase in healthcare costs, diagnostic delays and medico-legal disputes [3]. US is often wrongly considered as an imaging modality that needs short training, in the mistaken belief that it is, due to the widespread diffusion of the method, a very simple procedure to perform. Errors of ignorance are due to inadequate knowledge, whereas errors of implementation occur during the application of knowledge [3,4]_._ Furthermore, knowing the intrinsic US limits also represents at the same time the achievement of a good awareness of US imaging, the so-called “right measure”, so as to be able to better combine this imaging modality with others in order to achieve good diagnostic performance, and at the same time, make US a concrete diagnostic opportunity. The training and advanced study of US techniques, thorough knowledge of human anatomy, the study of US artifacts, and ultrasonographic semiotics are all crucial tools, and according to the increase in the supply of US performance and its clinical use in emergency scenarios, the need to implement universally accepted guidelines of US training courses appears imperative.

## 3. Errors of Interpretation

1. Bad artifacts;
2. Chest artifacts with no clinical context;
3. US-setting errors;
4. Anatomy and anatomical variants.

### 3.1. Bad Artifacts

Image artifacts are commonly found with US and can be confusing to the physician who encounters them. Some artifacts may be avoidable and result from an improper scanning technique. Other artifacts are generated by the physical limitations of the mode [5]. In clinical practice, side lobe artifacts, the mirror imaging effect, speed-displacement artifacts, refraction and reverberation artifacts, image-adaptation artifacts, and anisotropy are all common [5]. These are defined as follows:

#### 3.1.1. The Side Lobe Artifact

**Figure 1:**
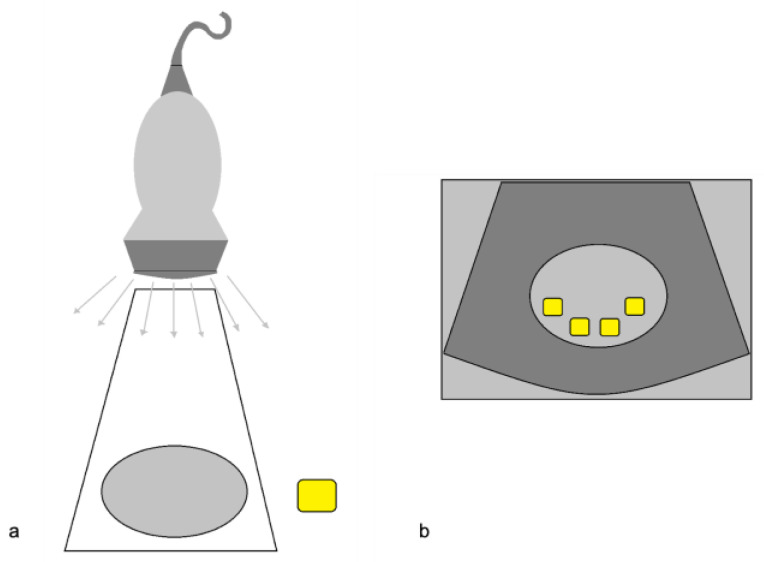
The side lobe artifact. (**a**) Diagram shows multiple beams outside the main US axis encountering an object (yellow box); (**b**) The display assumes that the return echoes that come from outside the main axis are mistakenly understood as coming from the main axis itself, and therefore misplaces and duplicates the structure (multiple yellow boxes) in the context of the target image. Modified from Feldman MK et al. [5].

A powerful reflecting surface settled out of the main US beam generates echoes that the transducer may detect (Figure 1).

**Figure 2:**
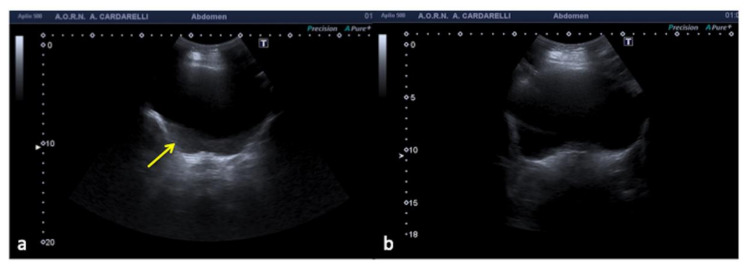
Transverse US image of filled bladder shows echoes (**a**, arrow) in the expected anechoic urine. Transverse US image obtained after optimal placement of the transducer (**b**) shows resolution of the intra-bladder echoes.

An example is given by the “pseudo-mud” which is observed at the bottom of the bladder or in the gallbladder (Figure 2). In this case, the correct setting of the US image, the focusing and the use of multiple scans allow the image quality to be improved [5,6].

#### 3.1.2. The Mirror-Imaging Effect

**Figure 3:**
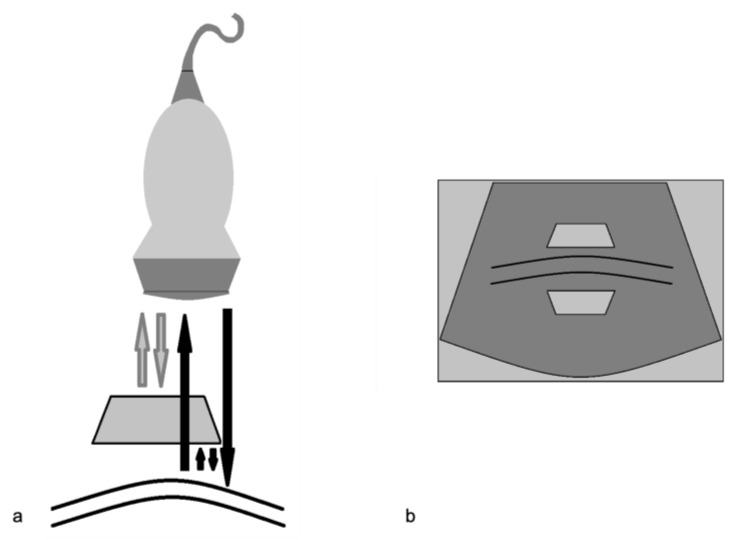
Mirror-image artifact. (**a**) In this diagram, the gray arrows represent the expected reflective path of the US beam. These echoes are displayed properly. The black arrows show an alternative path of the primary ultrasound beam. It encounters another structure (e.g., a nodular lesion) in its path and is reflected back to the highly reflective surface (e.g., diaphragm). It then reflects back towards the transducer again. (**b**) The echoes from the deeper reflective interface take longer to return to the transducer and are misplaced on the display. Modified from Feldman MK et al. [5].

**Figure 4:**
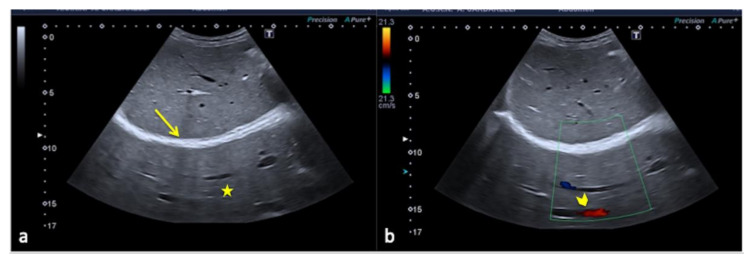
Oblique US image obtained at the level of the right hepatic lobe shows a solid-appearing structure posterior to the diaphragm (**a**, arrow) mimicking lung consolidation (**a**, star). The presence of a duplicated image of the hepatic vein (**b**, arrowhead) makes clear this is an artifact and not a real consolidation.

**Figure 5:**
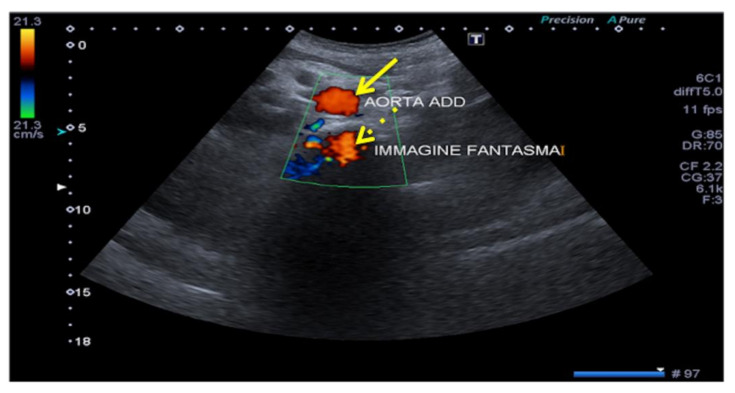
Aorta ghosting. Transverse mesogastric color-Doppler US image obtained at the level of the infra-renal aorta (arrow) shows aorta ghosting (dashed arrows) that projects backwards with the same color sign.

When the US of the primary incident beam passes through a curved interface with high specular reflection capacity (e.g., the diaphragm), the structure close to this interface can be reproduced on the US image both in its real position and in a symmetrical opposite position, beyond the reflecting surface (e.g., mirror image of the liver, which can be mistaken for a parenchymal consolidation, or the aorta, which can produce a “ghost image”) (Figure 3, Figure 4 and Figure 5). Mirror-image artifacts will disappear when the reflector is scanned with oblique orientation [5,6].

#### 3.1.3. The Speed-Displacement Artifact (Propagation Velocity Artifact)

**Figure 6:**
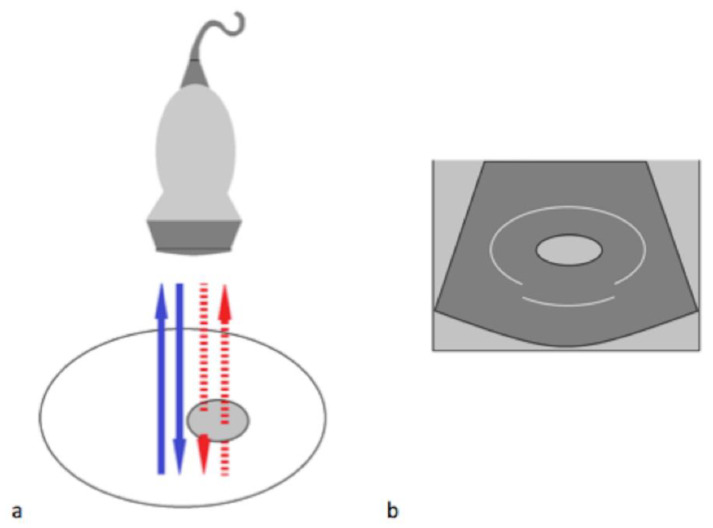
Speed displacement artifact. (**a**) In this diagram, the blue arrows represent the expected reflected path of the US beam. The red dashed arrows represent the path of an US beam that encounters an area of focal fat travelling slower than in the surrounding tissue. (**b**) Because the round trip of this echo is longer than expected, the posterior wall is displaced deeper on the display. Modified from Feldman MK et al. [5].

**Figure 7:**
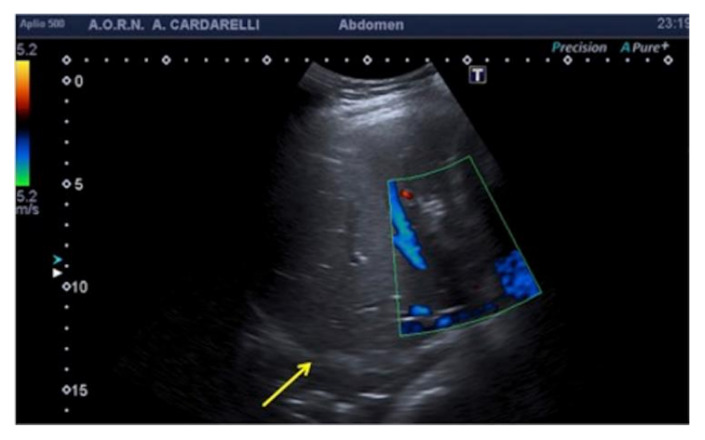
Longitudinal US image of the liver shows that the interface between the liver and the diaphragm (arrow) is discontinuous and focally displaced. This appearance may be explained by areas of focal fat within the liver.

This happens when the ultrasonic beam passes through the propagation medium at a much slower speed than expected (1540 m/s), causing the return echo to take longer to be decoded by the scan converter and to return (Figure 6 and Figure 7). The echoes thus appear deeper on the image than they really are due to the assumption that the length of time taken for a single round trip of an echo is related only to the distance that it travels [5]. The use of multiple scans can improve the image quality.

#### 3.1.4. Refraction Artifacts

**Figure 8:**
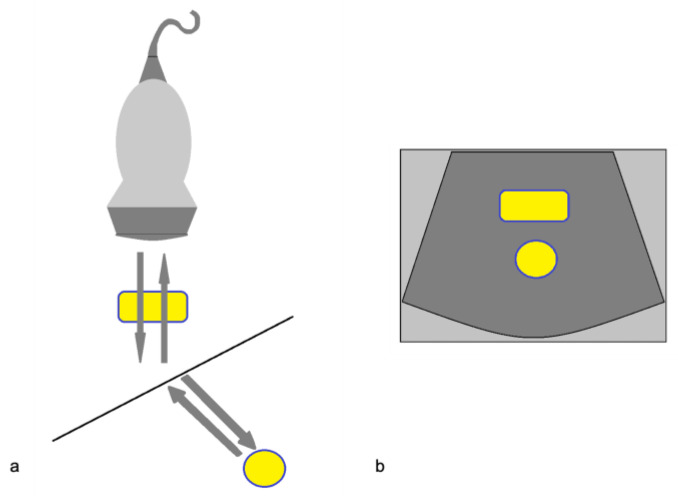
Refraction artifact. (**a**) Diagram shows the refraction or change in direction of the US beam due to an interface at a non-perpendicular angle. The difference in propagation speed between the two tissues can cause refraction to occur. (**b**) The object in the path of the refracted portion of the beam is misplaced because the processor assumes a straight path of the beam. Modified from Feldman MK et al. [5].

**Figure 9:**
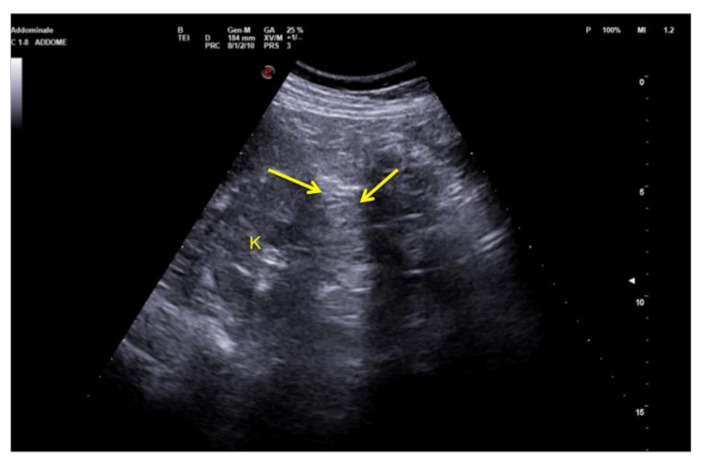
Coronal images of the left flank. Refraction of the US beam at the lower pole of the spleen causes apparent disruption of the of the middle third of the left kidney (arrows). K: kidney.

When the US beam encounters an interface that attenuates the sound to a greater or lesser extent than in the surrounding tissue, the strength of the beam distal to this structure will be either weaker or stronger than in the surrounding field [3]. This occurs because a focus material might cause the beam to deviate from its rectilinear path at an angle that causes a deflection towards the side with the higher resistance (Figure 8). Because this type of artifact can mimic a traumatic kidney rupture, it is recommended that scans are performed with coronal, axial, and sagittal pictures in order to overcome this interpretation deficiency (Figure 9) [5,6].

#### 3.1.5. Reverberation Artifacts

**Figure 10:**
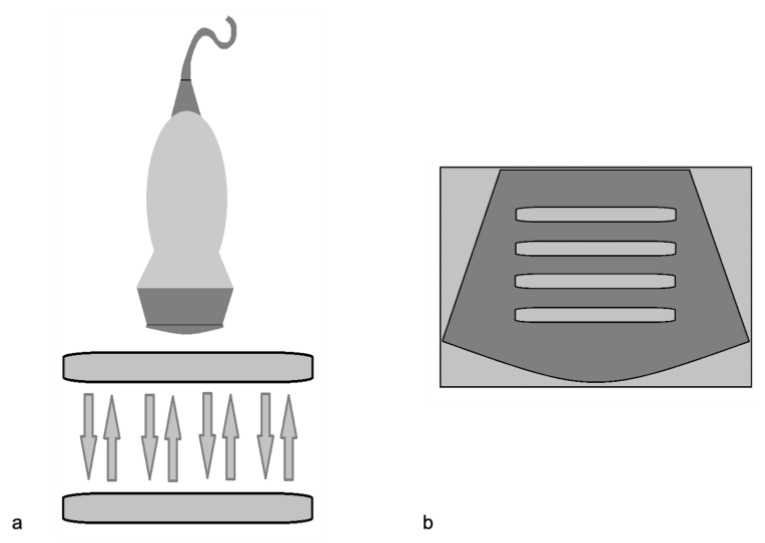
Reverberation artifact. (**a**) Diagram shows ultrasound echoes reflect back and forth between two highly reflective interfaces (“reverberates”) (**b**) The display shows multiple equally spaced signals extending into the deep field. Modified from Feldman MK et al. [5].

**Figure 11:**
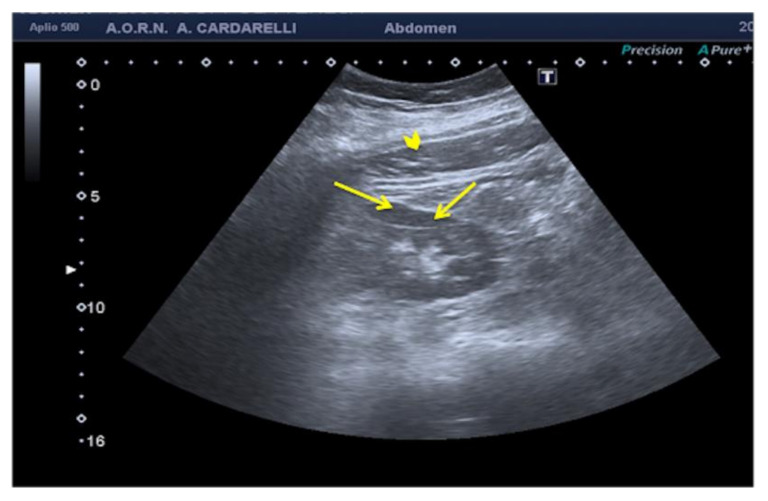
Coronal images of the right kidney. A reverberation artifact from strong echoes of the abdominal wall (arrowhead) projects over the lateral margin of the kidney, mimicking the presence of a subcapsular hematoma (arrows).

Because the US beam is reflected back and forth several times between two highly reflective parallel surfaces (“reverberates”), the US transducer interprets the reflected echoes as happening at deeper structures because they take longer to return to the transducer (Figure 10 and Figure 11) [3]. This very common mistake can be easily solved with a change of the angle of insonation in order to avoid the reverberation between strong parallel reflectors [5,6].

#### 3.1.6. Image-Adaptation Artifacts

**Figure 12:**
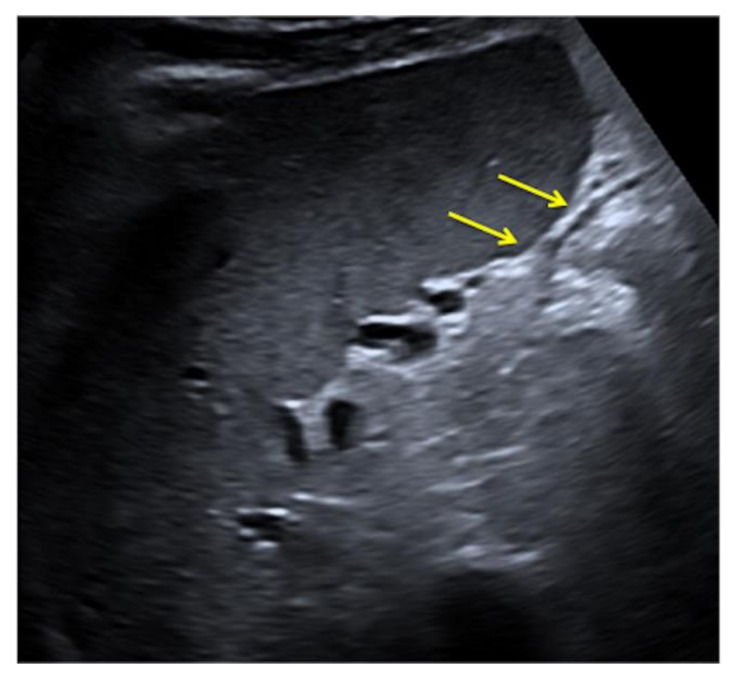
Pseudo-fluid produced by adaptive image processing artifact (arrows).

Real-time image processing methods have been developed to improve the contrast resolution of US images by focusing on texture details and reducing noise and artifacts [3]. Edge-enhancement filters, on the other hand, can produce an artifactual hypoechoic line parallel to a highly reflecting interface, simulating thin-fluid collection (Figure 12) [5,6].

#### 3.1.7. Anisotropy

**Figure 13:**
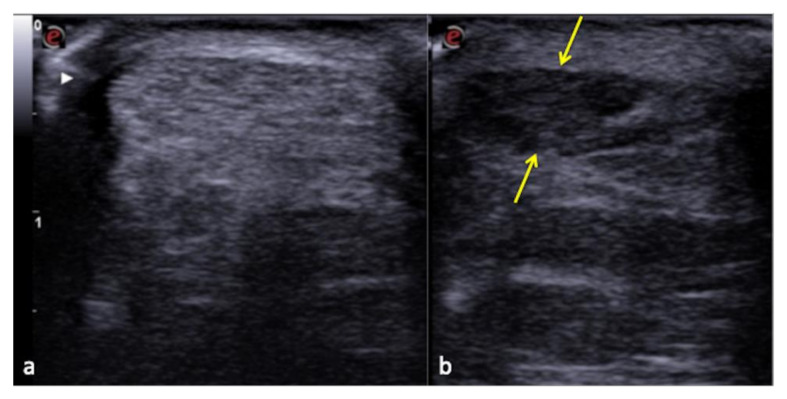
Normal Achilles tendon (**a**) and anisotropy-related artifact seen at the same tendon (**b**) that appears hypoechoic (arrows) due to an incorrect angle of the transducer.

In musculoskeletal applications, this is a common occurrence. It occurs when the US beam strikes a structure with a fibrillar structure (e.g., a ligament or tendon), causing the majority of the insonating sound beam to recede from the transducer. The fibrils have aberrant echogenicity, which the transducer interprets as a hypoechoic zone [5]. This anisotropic effect is dependent on the angle of the insonating beam (Figure 13). To overcome this, the insonation angle must always be perpendicular to the structures to be examined [7].

### 3.2. Chest Artifacts with No Clinical Context

Intrinsic or patient-related artifacts (such as subcutaneous emphysema), the coexistence of several pathologies that increase or decrease the sub-pleural air content (such as emphysema and atelectasis, respectively), or existing fibrotic interstitial lung disease, can all be confounding factors in the interpretation of lung US (LUS) findings in the acute setting, and are heavily influenced by the patient’s age [6]. LUS has intrinsic limits, and since it is based on the presence or absence of pulmonary artifacts such as A-lines or B-lines, it must be remembered that as well as recognizing them, they must necessarily be contextualized to the clinical data since they can have a dichotomous interpretation (Table 1) [8].

### 3.3. US-Setting Errors

**Figure 14:**
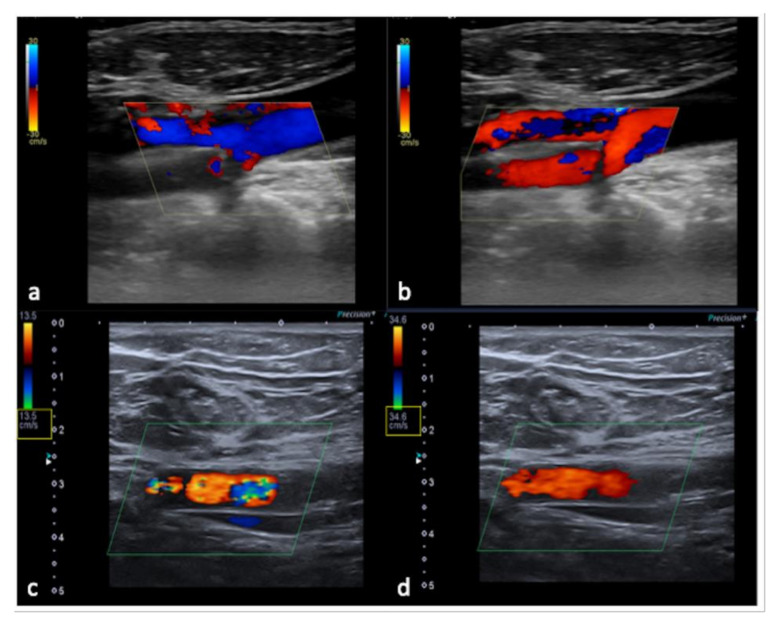
Flow sampling error with anomalous steering (**a**) misinterpreted as occlusion of the internal carotid artery; after steering correction (**b**) a normal artery patency is evident. False aliasing (**c**) and its correct setting (**d**) by changing the detection parameters upwards of the flow rate (yellow box, **c**,**d**).

**Figure 15:**
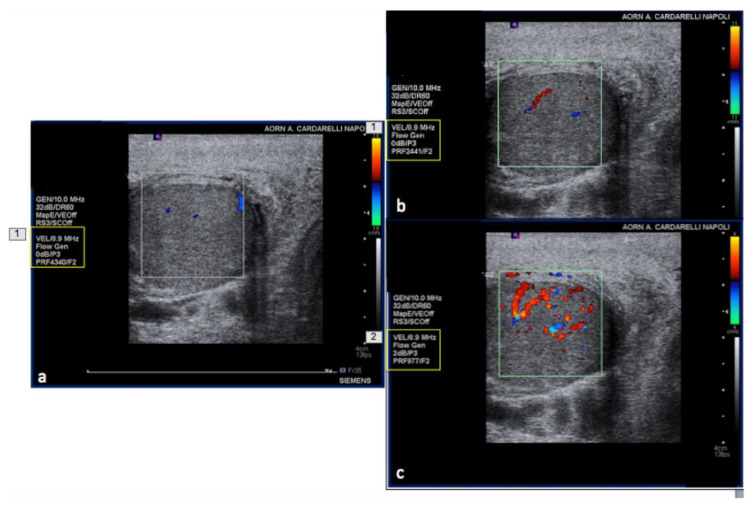
PRF setting. High- (**a**) middle- (**b**) and low- (**c**) PRF setting show progressive better evidence of intra-testicular flow at low setting (yellow box).

To avoid errors, a thorough understanding of the functionality and underlying mechanics of the US equipment is required. The goal should always be to produce the highest possible image quality by employing a check-list procedure to configure the correct system settings as well as the correct Doppler parameters, which are critical for interpreting clinical US findings (Table 2, Figure 14 and Figure 15) [6,9,10,11,12].

### 3.4. Anatomy and Anatomical Variants

**Figure 16:**
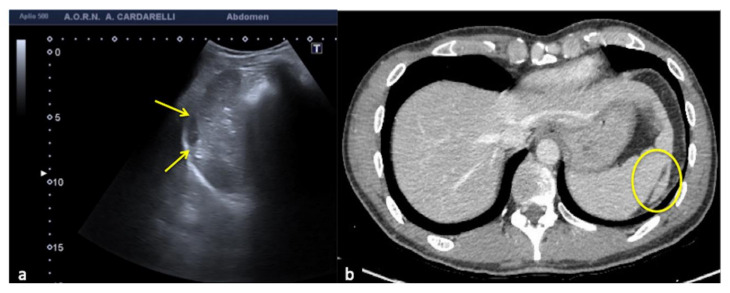
Coronal US scan of the left hypochondrium (**a**) shows a crescent-shaped hypoechoic area misinterpreted as hematoma (arrows) between the surface of the spleen and the left hemidiaphragm in a 25−year-old man investigated for trauma. On CT scan (**b**) it appears to be a hypertrophy of the left hepatic lobe with splenic kissing (circle).

**Figure 17:**
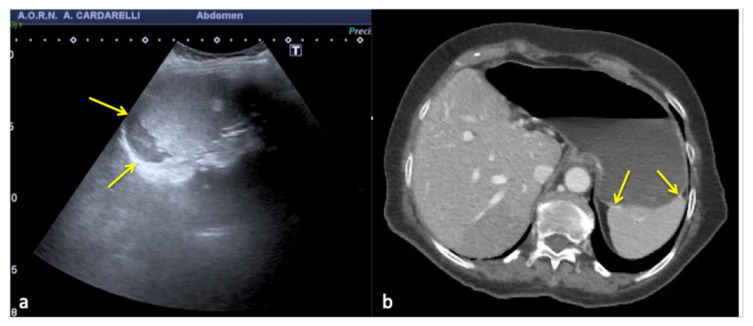
Transverse US image of the left hypochondrium (**a**) shows a large hypoechoic area misinterpreted as splenic hematoma (arrows) in a 31-year-old woman investigated for trauma. On CT scan (**b**) it appears to be a gastric fundus distended by fluid (arrows).

**Figure 18:**
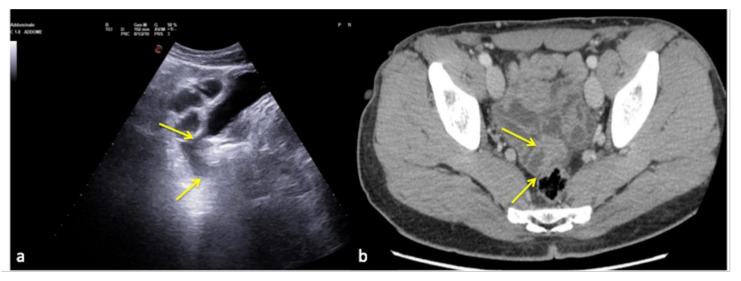
Longitudinal US pelvic scan (**a**) shows a small, triangular, hypoechoic area which may be misinterpreted as free fluid (arrows). On CT scan (**b**) it appears to be an intestinal loop (arrows).

**Figure 19:**
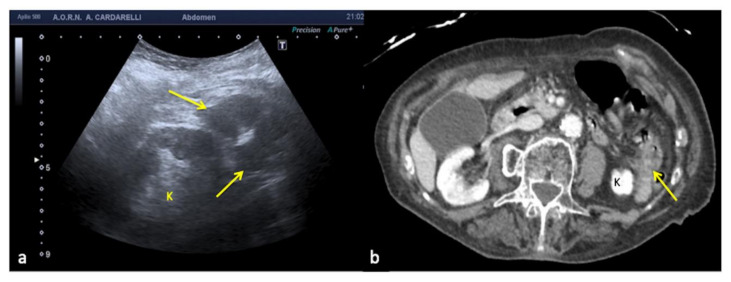
Longitudinal US left flank scan (**a**) shows at the lower pole of the kidney (K) a hypoechoic area which may be misinterpreted as retroperitoneal free fluid (arrows) in a 22-year-old man investigated for trauma. On CT scan (**b**) it appears to be a spastic intestinal loop (arrow).

**Figure 20:**
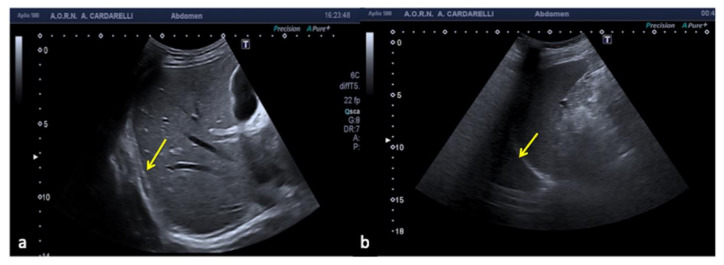
Subcostal US right approach (**a**) shows a hypo-anechoic elongated image (arrow) simulating a small right pleural effusion. Coronal US scan of the left hypochondrium (**b**) shows a mirror artifact that duplicates the image of the spleen (arrow) and mimics pleural effusion.

**Figure 21:**
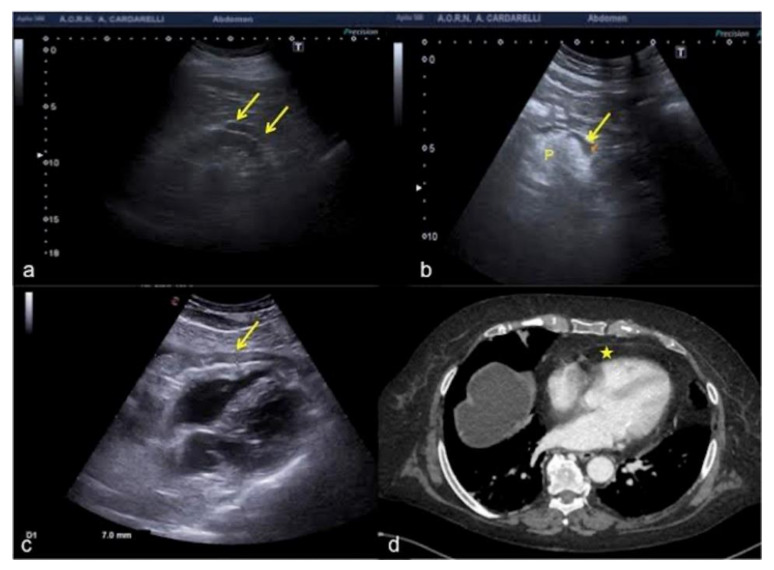
(**a**) Hypoechoic peri-renal fat, (**b**) peri-pancreatic fat and (**c**) pericardial fat misinterpreted as fluid collections (arrows). (**d**) On CT scan pericardial fat was clearly visible without any fluid collection (star) unlike what was wrongly diagnosed on US scan (**c**). P: pancreas.

**Figure 22:**
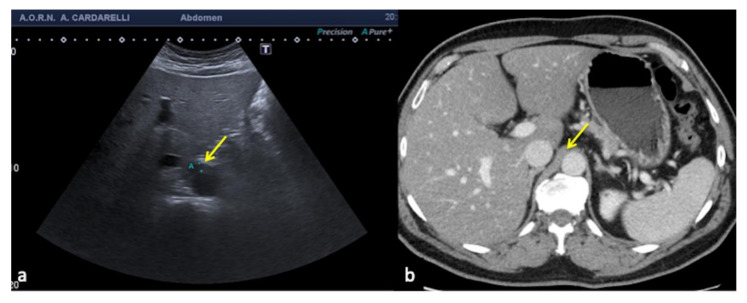
Transverse US scan of the epigastrium (**a**) shows a focal thickening of the aortic wall (arrow) misinterpreted as hematoma in a 45-year-old man investigated for high-dynamic deceleration trauma. On CT scan (**b**) it appears to be a hypertrophic right diaphragmatic pillar (arrow).

**Figure 23:**
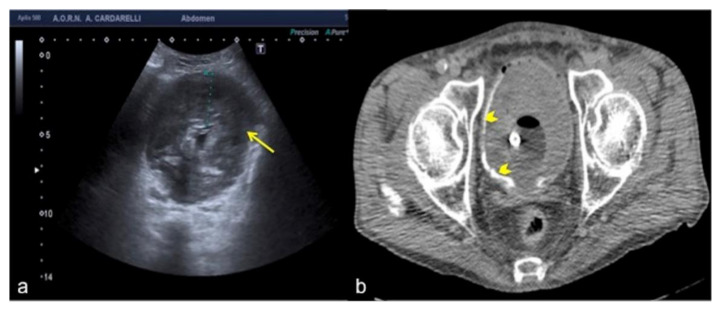
Transverse US scan of the bladder (**a**) shows a diffuse thickening of the wall (arrow) in a 78-year-old man investigated for hematuria. On CT scan (**b**) it appeared to be a large blood clot occupying the entire lumen of the bladder (arrowheads).

**Figure 24:**
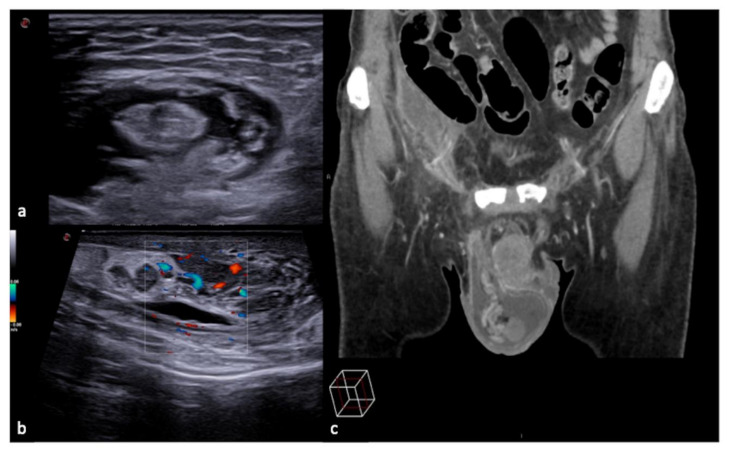
Longitudinal US B-mode (**a**) and color-Doppler (**b**) scan of the inguinal canal show a blockage of the inguinal canal misinterpreted as an inguinal hernia with congested intestinal loop. On CT scan (**c**) it appears to be a right epididymitis with funiculitis.

**Figure 25:**
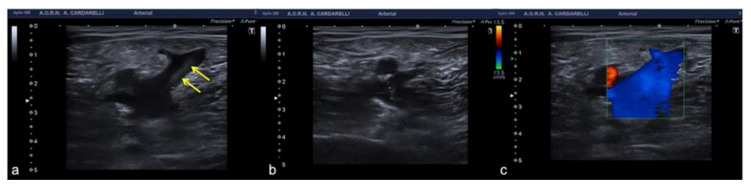
Rouleaux formation over the venous valves (**a**, arrows). After distal compression, the blood was squeezed and the rouleaux was finally cleared (**b**,**c**).

Some anatomical structures and variances might yield difficult-to-interpret pictures, which can lead to errors if not fully understood [6]. The most insidious in emergency situations are pseudo-splenic hematoma (Figure 16 and Figure 17), pseudo-collections of pleural, pericardial, peritoneal, and retroperitoneal fluids (Figure 18, Figure 19, Figure 20 and Figure 21), and pseudo-pneumothorax related to abolished lung sliding due to the lung pulse or to patient apnea [6,7]. Others, such as hypertrophy diaphragmatic pillar (Figure 22), bladder pseudo-masses (Figure 23), inguinal pseudo-hernias (Figure 24), and the rouleaux phenomena, are generic, random, and highly conditioned (Figure 25). Often it is the emergency condition of the clinical context itself as well as the traumatic accident that could influence interpretational doubts or over-diagnosis with the necessity of more diagnostic confirmations, involving more costly complex examinations, an increased waiting time for the final diagnosis and medico-legal disputes [3,13].

## 4. Errors of Underestimation

**Figure 26:**
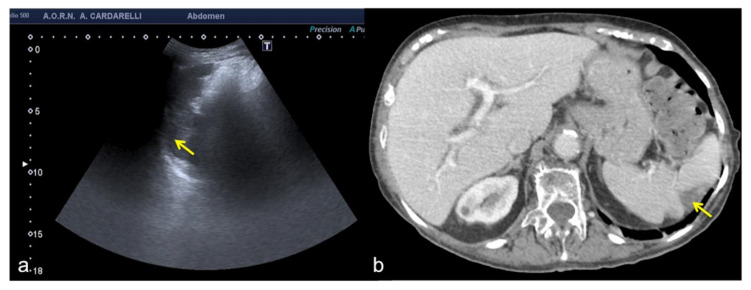
Coronal US scan of the left hypochondrium (**a**) shows partial exploration of the spleen with unrecognized traumatic injury (arrow) in a 22-year-old man investigated for trauma. On CT scan (**b**) it appears to be more evident (arrow).

This phenomenon depends on several items, such as the lack of a methodologically correct clinical approach, excessive diagnostic confidence generated by superficiality and/or lack of experience [3]. Due to partial visibility and poor attention or interest, these can damage the retroperitoneum or the spleen especially when carrying out focused assessment with sonography in trauma (FAST) examination (Figure 26).

**Figure 27:**
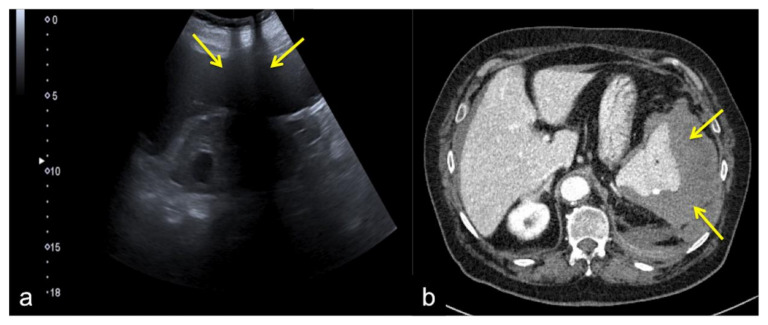
Coronal US scan of the left hypochondrium (**a**) is strongly influenced by the breakage of the probe crystals (arrows) and does not clearly show the large splenic hematoma. On CT scan (**b**) the splenic hematoma appears to be more evident (arrows).

Furthermore, poor image quality, as well as inconclusive and inadequate US reports, can often relate to errors of underestimation (Figure 27) [13,14,15].

It is important to document in detail the US findings found, as well as to provide for their archiving safeguarding in case of future medico-legal disputes. In the same way, an accurate and detailed report emphasizing the importance of the description of pathological changes detected should not be ambiguous, but easy to understand and timely with respect to the clinical question [3].

### How to Avoid Diagnostic Errors in Ultrasound: Is Artificial Intelligence the Right Way?

In this new era of cutting-edge medical advances, artificial intelligence (AI) has emerged as a subset of computer science involved in human-like processes such as learning, adapting, and solving complex problems. The current branches of AI in medical imaging mainly include machine learning (ML) and deep learning (DL), consisting of algorithms that can make predictions or decision tasks without prior explicit programmed rules [15]. ML algorithms use iterative statistical learning methods from “training” data to progressively improve the model performance over time, enabling recognition patterns in large datasets and classification of instances [16]. DL is a subgroup of ML techniques, structured as artificial neural networks which consist of multi-layered networks that automatically extract features without prior labels and perform high-level tasks [17]. The application of AI models coupled to medical imaging has been embraced in US image analysis to empower the clinical–radiological workflow and to reduce US errors derived by different image variation factors such as operator-, scanner-, and patient-dependent. Of note, the development of AI-based US should assist less-experienced users with performing correct examinations, ultimately improving the clinical decision process. In this regard, recent evidence has shown image-quality improvement by using AI algorithms to enhance resolution, making the US scans both more detailed and easier to read [18]. Furthermore, AI systems have been applied to specific US image-based tasks such as disease classification, abnormality detection, image segmentation, and prognosis assessment, and for different organ systems including thyroid, breast, abdomen and pelvis, obstetrics and gynecology, heart, and the musculoskeletal system [19]. Although more clinical evidence is still necessary, the outlook of AI in US imaging remains bright.

## 5. Conclusions

US quality is operator-dependent and subjective to interpretive error. Following specific rules concerning technique and interpretation and always integrating emergency US findings into the broader clinical context could avoid many misdiagnoses and thus enhance patient safety. Knowledge of common sites of artifacts and their typical/atypical appearances will help to prevent misinterpretation of these findings and may lead to improved diagnostic accuracy. Furthermore, the development of artificial intelligence (AI)-based US represents a useful additional tool of imaging modality. This aids the clinical decision process, making US scans both more detailed and easier to read. This article discussed the most common and uncommon diagnostic errors and mistakes in US examinations. Similarly to other diagnostic tools, it is a fact that errors may occur in the emergency room. Therefore, whether we are talking about common or intrinsic errors rather than uncommon dynamic or variable errors, the risk of a mistake remains. Besides that, the increased complexity in the interpretation of dynamic errors emphasizes the importance of proper training and credentialing for the operator. It is, however, understood that, beyond any technological progress, with a knowledge of the intrinsic limits of US and of its potential “bad” artifacts, as well as a standardized US approach, mistakes can be avoided. This helps to safeguard patient health and prevent potential medico-legal disputes associated with such mistakes.

## Figures and Tables

**Table 1:** LUS artifact dichotomy. Modified from Di Serafino et al. [8].

A-Lines		B-Lines	
Normal	Abnormal	Normal	Abnormal
Healthy patients	Atelectasis Asthma Chronic obstructive pulmonary disease (COPD) Pneumothorax	Normal finding	Degrees of interstitial disease Pneumonia Pneumothorax (possible)

**Table 2:** Steps to achieve optimal settings for B-Mode and Doppler US. Modified from Zander D et al. [9].

Steps	B-Mode Parameter	Remarks
1st	Transmission power	Depth penetration is improved and scattering is reduced with increasing transmission power.
2nd	Gain	Adjust as low as possible to avoid overexposure. The adjustment is almost always performed during the execution of an exam to reduce some artifacts and increase the visibility of low-contrast sound formations with respect to the surrounding tissues. By selectively amplifying the echoes from greater depths, using the time-gain compensation (TGC) method or depth-gain compensation (DGC), equal reflectors at unequal depths are displayed as structures of equal brightness on the monitor.
3rd	Frequency	Pivotal element of ultrasound image quality. Adjustment can be of great help with both convex and linear transducers. This parameter affects the spatial resolution and mainly the axial resolution.
4th	Depth penetration	Adjust to the structure of interest. The adjustment is almost always carried out during the execution of an exam, as it is important in US to start from the greatest possible spatiality and then move on to the detailed analysis.
5th	Focal zone(s)	At the level of interest or use focus. This parameter optimizes the lateral resolution.
6th	Further settings	Only in the case of insufficient image quality: change the pre-set, adjust the dynamic range, grey maps, persistence, and/or frame rate.
**Steps**	**Color and Pulse Doppler Parameter**	**Remarks**
1st	Pulse repetition frequency (PRF)	Optimal PRF proportional to the flow to be studied and in principle must be lowered until it almost reaches the aliasing threshold.
2nd	Size and position of the color box	The color box must be neither too wide, too small, too long nor too deep.
3rd	Angle correction	To calibrate the velocity scale for the angle between the US beam and the blood flow being measured. Ideally, the direction of flow should be at an approximately 45–60° angle relative to the transducer.
4th	Steering	Use probe positioning/beam steering to obtain satisfactory beam/vessel angle
5th	Further settings	Wall filters; inversion of flow; color gain; spectral gain; baseline.

## Data Availability

Data sharing is not applicable.
